# Conformational flexibility in the zinc solute-binding protein ZnuA

**DOI:** 10.1107/S2053230X22001662

**Published:** 2022-02-28

**Authors:** Elsie Laban Yekwa, Fred Allen Serrano, Erik Yukl

**Affiliations:** a Department of Chemistry and Biochemistry, New Mexico State University, 1175 North Horseshoe Drive, Las Cruces, NM 88003, USA

**Keywords:** zinc, ZnuA, *Citrobacter koseri*, solute-binding proteins, ATP-binding cassette transporters

## Abstract

Zinc is an essential metal for all kingdoms of life, making its transport across the cell membrane a critical function. In bacteria, high-affinity zinc import is accomplished by ATP-binding cassette (ABC) transporters, which rely on extracellular solute-binding proteins (SBPs) of cluster A-I to acquire the metal and deliver it to the membrane permease. These systems are important for survival and virulence, making them attractive targets for the development of novel antibiotics. *Citrobacter koseri* is an emerging pathogen with extensive antibiotic resistance. High-affinity zinc binding to the *C. koseri* cluster A-I SBP ZnuA has been characterized and the structure of the zinc-bound (holo) form has been determined by X-ray crystallography. Remarkably, despite 95% sequence identity to the ZnuA homologue from *Salmonella enterica*, *C. koseri* ZnuA exhibits a different zinc-coordination environment and a closed rather than an open conformation. Comparison with structures of another close ZnuA homologue from *Escherichia coli* suggests a surprisingly flexible conformational landscape that may be important for efficient zinc binding and/or delivery to the membrane permease.

## Abstract 2

In bacteria, high-affinity zinc import is accomplished by ATP-binding cassette (ABC) transporters, which rely on extracellular solute-binding proteins (SBPs) to acquire the metal and deliver it to the membrane permease. The crystal structure of the zinc-bound (holo) form of *Citrobacter koseri* SBP ZnuA has been determined. Despite 95% sequence identity to the ZnuA homologue from *Salmonella enterica*, *C. koseri* ZnuA exhibits different zinc-coordination and a closed rather than open conformation. Comparison with other close ZnuA homologue structures suggests a flexible conformational landscape that may be important for zinc binding and/or delivery to the permease.

## 1. Introduction

The ATP-binding cassette (ABC) transporters are a superfamily involved in the translocation of numerous substrates across the cell membrane using ATP hydrolysis (Higgins, 1992 ▸). In addition to the membrane permease and cytoplasmic ATPase, bacterial importers of this type also require a solute-binding protein (SBP) in the periplasm or outer membrane (Davidson *et al.*, 2008 ▸; van der Heide & Poolman, 2002 ▸) to specifically and efficiently bind and deliver the substrate (Khare *et al.*, 2009 ▸). Upon binding to the cognate substrate, the SBP is generally thought to convert from an open to a closed conformation, which is important for recognition by the membrane permease and subsequent transport (Davidson *et al.*, 2008 ▸; Quiocho & Ledvina, 1996 ▸). However, the degree of conformational change varies dramatically among different SBPs and substrates, with some exhibiting very little change (Berntsson *et al.*, 2010 ▸). Further, recent solution work has shown that dynamic interconversion between an ensemble of open and closed states is important for the specific transport of cognate substrates (de Boer *et al.*, 2019 ▸). Thus, a simple two-state mechanism for permease recognition and cognate substrate transport is likely to be an oversimplification for many ABC transporters.

SBPs have been classified into seven clusters (A–G) based on structure, with further subdivisions based on substrate specificity (Berntsson *et al.*, 2010 ▸, Scheepers *et al.*, 2016 ▸). By this classification, SBPs that bind zinc, manganese or iron belong to cluster A-I, which is defined by two related α/β domains linked by a long α-helix. Within this cluster, sequence similarity identifies several groups that differ on the basis of substrate specificity, coordination environment and the presence of a long flexible loop region near the substrate-binding site (Loisel *et al.*, 2008 ▸). The ZnuA proteins of Gram-negative species primarily belong to group II, which is defined by zinc specificity, the presence of a long flexible loop rich in His and Asp/Glu residues, and metal coordination by three His residues and one Glu residue or water in a tetrahedral geometry. Deletion mutants show diminished infection potential and virulence in infection models for several human pathogens including *Salmonella enterica* (Ammendola *et al.*, 2007 ▸), *Vibrio cholerae* (Sheng *et al.*, 2015 ▸), *Brucella abortus* (Yang *et al.*, 2006 ▸) and *Acinetobacter baumanii* (Hesse *et al.*, 2019 ▸), highlighting the potential of these proteins as antibiotic drug targets. Indeed, a lead compound was identified that bound to ZnuA from *S. enterica* and inhibited the growth of this organism (Ilari *et al.*, 2016 ▸). However, to our knowledge this was never developed for clinical use. While the early indications are promising, the effective exploitation of zinc solute-binding proteins will likely require detailed structural and mechanistic data.

Several crystal structures of cluster A-I SBPs in group II have been solved, including some in both holo and apo states. ZnuA from *Escherichia coli* (*Ec* ZnuA) shows an open conformation in the apo state, and zinc binding elicits conversion to the closed state primarily through motions of the C-terminal domain (CTD), with the zinc coordinated by Glu59, His60, His143 and His207 (Yatsunyk *et al.*, 2008 ▸; Li & Jogl, 2007 ▸). The holo structure of *Synechocystis* ZnuA is also closed, but the co­ordinating Glu residue is not conserved and a water molecule occupies the fourth coordination site (Banerjee *et al.*, 2003 ▸). Finally, holo *S. enterica* ZnuA (*Se* ZnuA) exhibits an open conformation and His60 is replaced by a residue from the flexible loop, His140 (Ilari *et al.*, 2011 ▸). Apo structures of deletion mutants of the flexible loops for the latter two proteins are in closed conformations (Wei *et al.*, 2007 ▸). Thus, there appears to be significant flexibility in coordination environment and conformation among the ZnuA proteins, which may reflect dynamics that are important for substrate specificity and permease recognition.

Here, we characterize zinc binding to the cluster A-I SBP ZnuA from the human pathogen *Citrobacter koseri* (*Ck* ZnuA) and present the crystal structure of the holo form. *C. koseri* is a Gram-negative organism that is associated with an increasing number of primarily nosocomial infections. Further, the incidence of multidrug resistance in *Citrobacter* species is widespread and rising, including resistance to the last-resort antibiotic class of carbapenems (Yao *et al.*, 2021 ▸). This severely limits treatment options and highlights the importance of identifying new antibiotic drug targets in this organism. *Ck* ZnuA is highly homologous to ZnuA proteins from *S. enterica* and *E. coli*, with 95% and 85% sequence identity, respectively, excluding the periplasmic targeting sequences. Despite this, these proteins exhibit a remarkable diversity of structures and metal-coordination environments between them, allowing us to present an ensemble of structures in both apo and holo forms. The results indicate a surprisingly plastic zinc-binding environment for the ZnuA family.

## 2. Materials and methods

### 2.1. Macromolecule production

The intact gene encoding wild-type (WT) *Ck* ZnuA (UniProt ID A8AFI6) was amplified by PCR from *C. koseri* strain ATCC BAA-895 genomic DNA and cloned into pCDFDuet (Novagen) at NdeI and Acc65I/KpnI restriction sites. The ZnuA loop deletion lacking residues 124–143 (ΔLoop ZnuA) was generated using the Q5 Site-Directed Mutagenesis Kit (New England BioLabs). Both plasmids were confirmed by sequencing. Both WT and ΔLoop ZnuA were expressed in *E. coli* BL21(DE3) cells grown in LB medium containing 50 µg ml^−1^ streptomycin at 37°C with shaking at 250 rev min^−1^ to an OD_600_ of 0.8–1.0. Overexpression was induced by isopropyl β-d-1-thiogalactopyranoside at a concentration of 1.0 m*M* and the temperature was decreased to 20°C. After overnight growth with shaking, the cells were harvested by centrifugation at 4000*g* for 30 min at 4°C.

The periplasmic fraction was prepared using an osmotic shock protocol adapted from Wang *et al.* (2003 ▸). Polyethylamineimine (PEI) was added to 0.5%(*v*/*v*) and centrifuged at 25 000*g* at 4°C for 20 min. Ammonium sulfate was added to the supernatant to 70% saturation and centrifuged as above for 30 min. The ammonium sulfate pellet was then drained and resolubilized in 20 m*M* Tris pH 8.0 and initially purified at this pH by anion-exchange chromatography (IEC) using a HiTrap Q HP column (Cytiva) with an increasing gradient of NaCl. ΔLoop ZnuA was dialyzed against 20 m*M* Tris pH 9.0 and IEC was performed at this pH. Fractions containing *Ck* ZnuA were combined, concentrated to <1 ml and further purified using a HiPrep Sephacryl S-200 HR size-exclusion chromatography (SEC) column (Cytiva) equilibrated with 50 m*M* Tris pH 8.0, 150 m*M* NaCl. After final purification by SEC, all proteins were highly pure as judged by SDS–PAGE. WT *Ck* ZnuA and ΔLoop *Ck* ZnuA concentrations were determined using extinction coefficients at 280 nm of 31 162 and 28 816 *M* ^−1^ cm^−1^, respectively, calculated as described previously (Edelhoch, 1967 ▸). Macromolecule-production information is summarized in Table 1 ▸.

#### 2.1.1. Generation of apoproteins and metal quantitation

Apoproteins were generated by dialysis at 4°C and complete zinc removal was confirmed by inductively coupled plasma–optical emission spectrometry (ICP-OES) as described previously (Neupane *et al.*, 2019 ▸). Buffer blanks were generated identically to protein samples using an equal volume of buffer relative to protein solution. Metal content was quantified using a Perkin–Elmer 2100 DV ICP-OES calibrated with a multi-element standard (Alpha Aesar) at a wavelength of 213.857 nm for zinc.

#### 2.1.2. Mag-Fura-2 competition assay

Zinc-binding affinities were measured using a Mag-Fura-2 (MF-2) competition assay derived from Golynskiy *et al.* (2006 ▸) using concentrations and instrument parameters as described previously (Neupane *et al.*, 2019 ▸; Handali, Neupane *et al.*, 2015 ▸; Handali, Roychowdhury* et al.*, 2015 ▸). Experiments were performed in triplicate and the data were fitted using *DYNAFIT* (Kuzmič, 1996 ▸, 2006 ▸, 2009 ▸) and a script modified from that described previously (Handali, Neupane *et al.* 2015 ▸) to account for three zinc-binding sites in the protein. *DYNAFIT* uses simultaneous nonlinear algebraic equations to fit equilibrium data rather than a single binding isotherm expression. For ΔLoop *Ck* ZnuA, the calculated *K*_d_ values for sites 2 and 3 were greater than 100-fold that of MF-2, indicating that multiple metal binding could not be detected using this assay, and an equivalent fit was acquired using a single binding site. The affinity of MF-2 for zinc in our buffer system was determined prior to each series of experiments and the *K*_d_ varied from 59 to 110 n*M*.

### 2.2. Crystallization

Initial crystallization hits for WT *Ck* ZnuA were identified using the Hauptman–Woodward Institute standard screen (Luft *et al.*, 2004 ▸). These were optimized in-house and diffraction-quality crystals were grown by vapor diffusion using a 1:1 ratio of 18 mg ml^−1^ *Ck* ZnuA reconstituted with one equivalent of zinc and precipitant solution consisting of 0.1 *M* sodium citrate pH 6.0, 0.05 *M* ammonium acetate, 1 m*M* hexatungstotellurate (TEW; Mauracher *et al.*, 2014 ▸) incubated at 298 K. TEW was synthesized in-house as described previously (Mauracher *et al.*, 2014 ▸). The crystals were cryoprotected in reservoir solution containing 10% PEG 400 and were cryocooled in liquid nitrogen. Crystallization information is summarized in Table 2 ▸.

### 2.3. Data collection and processing

Diffraction data were collected at 100 K on beamline 5.0.1 at the Advanced Light Source at Berkeley National Laboratory, indexed and integrated with *XDS* version 0.92 (Kabsch, 2010*a* ▸,*b* ▸) and scaled using *AIMLESS* (Winn *et al.*, 2011 ▸). Data-collection and processing statistics are summarized in Table 3 ▸.

### 2.4. Structure solution and refinement

The model of zinc-bound *Se* ZnuA (PDB entry 2xqv; Ilari *et al.*, 2011 ▸) lacking zinc and waters was used as the search model for molecular replacement using *Phaser-MR* (McCoy *et al.*, 2007 ▸). Manual model building was performed in *Coot* (Emslet *et al.*, 2010 ▸) and further rounds of refinement were performed in *REFMAC* (Murshudov *et al.*, 2011 ▸). Atomic coordinates of zinc-bound *Ck* ZnuA have been deposited in the PDB as entry 7rcj. Figures were prepared using *PyMOL* (http://www.pymol.org), which was also used for pairwise structural alignments of *Ck* ZnuA with other solute-binding proteins. Refinement statistics are summarized in Table 4 ▸.

## 3. Results and discussion

### 3.1. Zinc binding to *C. koseri* ZnuA

**Figure 1:**
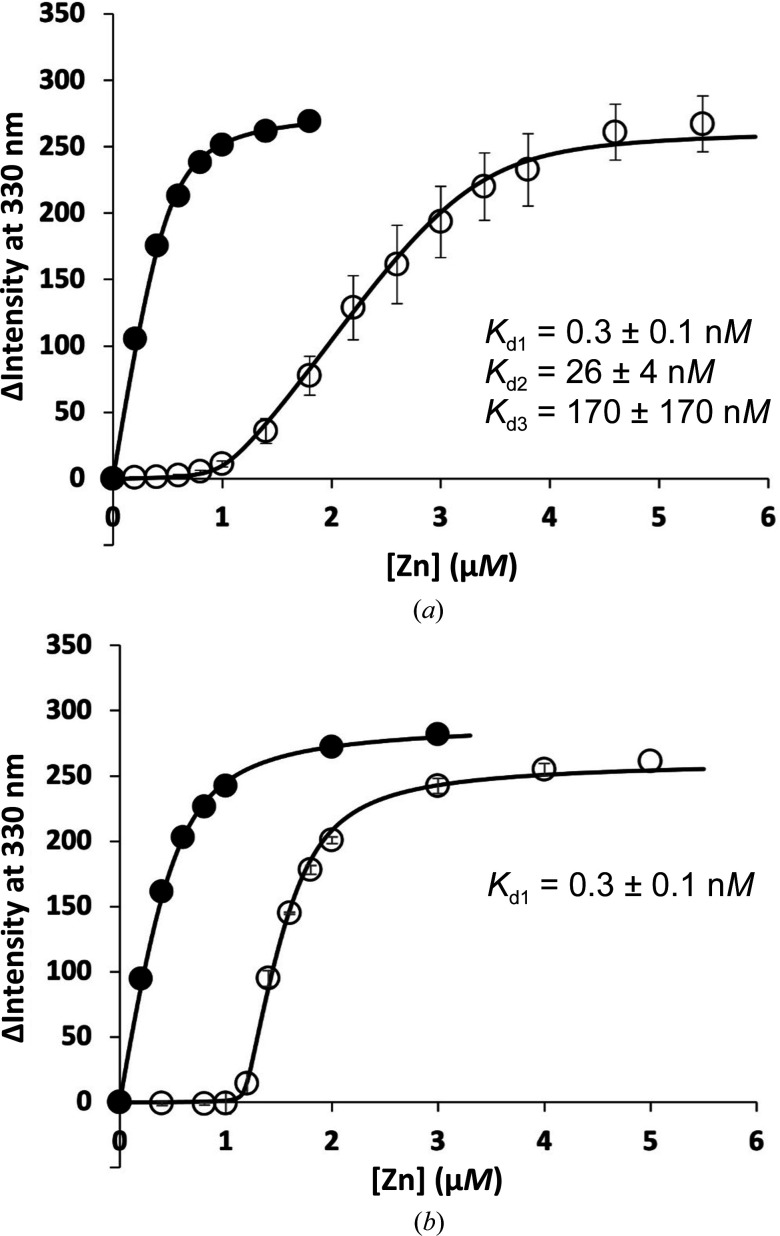
Zinc binding by *C. koseri* ZnuA determined by MF2 competition assay. The intensity change at 330 nm with increasing zinc in the absence (solid circles) and presence (empty circles) of protein is shown for (*a*) apo WT *Ck* ZnuA and (*b*) ΔLoop *Ck* ZnuA. Titrations were performed in triplicate and error bars represent the standard deviation between experiments. Fits are shown as solid lines. Dissociation constants ± standard deviations (*n* = 3) are indicated.

Since *Ck* ZnuA had not previously been characterized, the affinity and stoichiometry of zinc binding were determined using a competitive fluorescence assay with Mag-Fura-2 (MF2; Golynskiy *et al.*, 2006 ▸; Fig. 1 ▸). We also investigated binding to a deletion mutant lacking a flexible, His-rich loop (residues 124–143, ΔLoop *Ck* ZnuA) common to most zinc-specific SBPs. The results show that *Ck* ZnuA binds at least three zinc ions with high affinity. The observation that ΔLoop *Ck* ZnuA binds only a single zinc ion indicates that the formation of two additional zinc-binding sites requires the presence of the His-rich loop. The large error on the lowest-affinity binding event is a consequence of weak binding affinity relative to the MF2 probe. Nevertheless, this assay confirms high-affinity zinc binding consistent with that observed for other cluster A-I SBPs.

### 3.2. Crystal structure of *C. koseri* ZnuA

**Figure 2:**
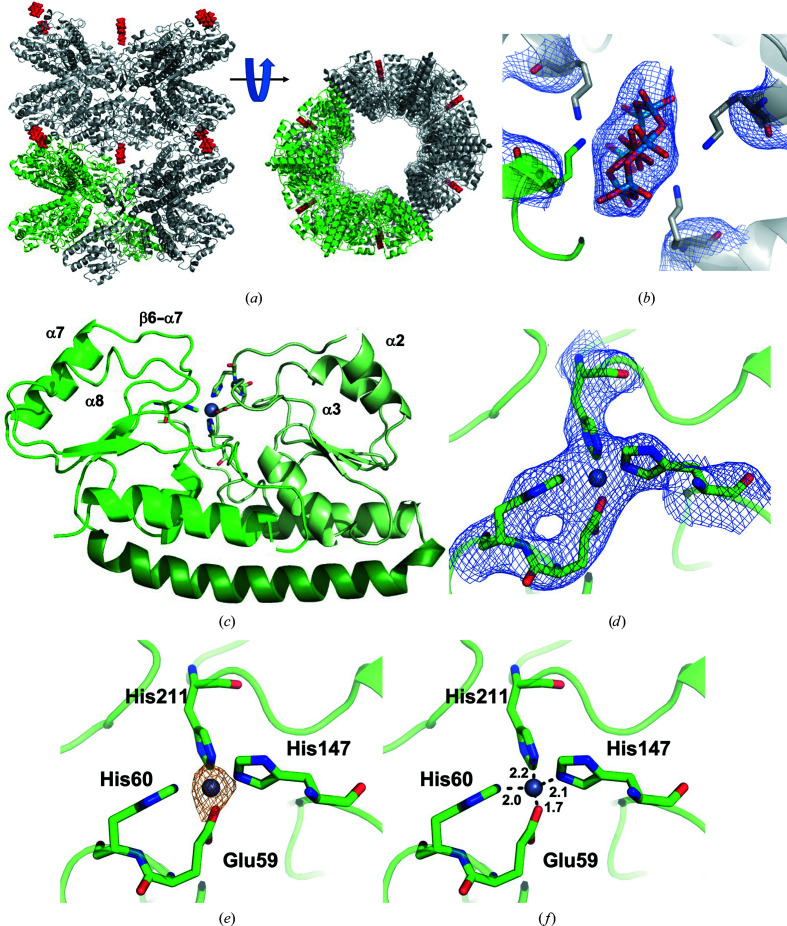
The crystal structure of holo *Ck* ZnuA. (*a*) The lattice of the *Ck* ZnuA crystals. The protein is shown in ribbon form with one asymmetric unit shown in green and the remaining three in gray. TEW is shown as red spheres. (*b*) TEW binding site showing TEW as sticks colored according to element (oxygen, red; tungsten, blue; tellurium, orange) and Lys91 from each of four symmetry-related protein molecules as sticks. 2*F*_o_ − *F*_c_ electron density is shown as a blue mesh contoured at 1.0σ. (*c*) Whole protein shown as a ribbon diagram with the NTD in light green, the linker helix in dark green and the CTD in medium green, with zinc shown as a small gray sphere and ligands shown as sticks colored by element. (*d*) The zinc-binding site showing 2*F*_o_ − *F*_c_ electron density contoured at 1.5σ, (*e*) an anomalous difference map contoured at 3.0σ and (*f*) zinc–ligand distances in Å.

Initial crystallization conditions yielded small, disordered crystals with poor diffraction. A screen of additives identified hexatungstotellurate (TEW; Mauracher *et al.*, 2014 ▸) to contribute to larger, better-ordered crystals, as has previously been reviewed (Bijelic & Rompel, 2017 ▸, 2018 ▸). These crystals belonged to space group *P*2, with six copies of *Ck* ZnuA in the asymmetric unit. Three copies of TEW are also evident at special positions, demonstrating how they are able to generate crystal contacts and stabilize the crystal lattice (Fig. 2 ▸ *a*). With a formal charge of minus six, TEW often interacts electrostatically with positively charged residues and through hydrogen bonding in protein structures (Mac Sweeney *et al.*, 2018 ▸; Vandebroek *et al.*, 2020 ▸; Bijelic *et al.*, 2015 ▸; Sobala *et al.*, 2020 ▸). Each TEW in the *Ck* ZnuA structure is in close proximity to Lys91 of four symmetry-related protein chains (Fig. 2 ▸ *b*). However, poor electron density around the side chains precludes the confident assignment of these residues as coordinating ligands.

Even with the stabilizing interactions provided by TEW, the resolution of the data was relatively poor, prompting us to use the more generous resolution cutoff based on CC_1/2_ (Karplus & Diederichs, 2012 ▸, 2015 ▸; Table 3 ▸). The resulting electron-density map was of good quality throughout, and the *R* factors for the refined structures are relatively low, illustrating the utility of this approach. The average *B* factors are unusually high, which is likely to be a consequence of the low resolution of the data and the relatively high solvent content (∼64%) of these crystals. Finally, there are a relatively large number of Ramanchandran outliers, which are not typically observed in other ZnuA structures. Several of these are localized to helix α8 and are flagged as outliers in each of the six protein copies (for example residues 266, 271 and 272). While this suggests that these may actually exist as outliers, the low resolution of the data preclude us from ascribing any functional significance to this observation.

The overall structure of *Ck* ZnuA is similar to those of other cluster A-I SBPs solved to date and consists of an N-terminal domain (NTD) and a C-terminal domain (CTD) connected by a long α-helix (Fig. 2 ▸ *c*). It is in the closed conformation with the β6–α7 loop closed over the zinc site. All residues could be modeled into the electron density with the exception of the His-rich loop, which is typical of cluster A-I SBP structures, where this region is disordered. In all six chains zinc is bound between the domains and is coordinated by Glu59, His60, His147 and His211 (Fig. 2 ▸ *d*). This is the same coordination environment as is observed in the *Ec* ZnuA (Yatsunyk *et al.*, 2008 ▸; Li & Jogl, 2007 ▸) and *Synechocystis* ZnuA (Banerjee *et al.*, 2003 ▸) structures, but differs from that observed for *Se* ZnuA, in which one of the flexible-loop His residues (His140) replaces His60 (Ilari *et al.*, 2011 ▸). An anomalous difference map confirms the position of zinc in this structure (Fig. 2 ▸ *e*), which is bound in a tetrahedral geometry (Fig. 2 ▸ *f*).

### 3.3. Differences and similarities between ZnuA proteins

Like many ZnuA homologues, *Ck* ZnuA is capable of binding multiple zinc ions, which requires the presence of the His-rich loop (Yatsunyk *et al.*, 2008 ▸; Ilari *et al.*, 2011 ▸, 2014 ▸; Pederick *et al.*, 2015 ▸; Desrosiers *et al.*, 2007 ▸; Lu *et al.*, 1997 ▸). Although conserved in many zinc-specific cluster A-I SBPs, the function of this loop remains enigmatic. A function as a zinc chaperone to the high-affinity site has been proposed (Banerjee *et al.*, 2003 ▸; Desrosiers *et al.*, 2007 ▸; Lu *et al.*, 1997 ▸), as has a regulatory function as a sensor of high zinc concentrations (Wei *et al.*, 2007 ▸). However, little conclusive evidence has been provided for either case. Most of the sequence differences between the ZnuA proteins from *C. koseri* (*Ck* ZnuA), *E. coli* (*Ec* ZnuA) and *S. enterica* (*Se* ZnuA) are found in this region. The *Ck* ZnuA loop allows the binding of two additional zinc ions, while only one zinc-binding event could be ascribed to the loop in *Ec* ZnuA (Yatsunyk *et al.*, 2008 ▸) and *Se* ZnuA (Ilari *et al.*, 2014 ▸). This may be a simple consequence of the presence of two extra His residues in the *Ck* ZnuA loop relative to the other ZnuA homologues discussed here.

**Figure 3:**
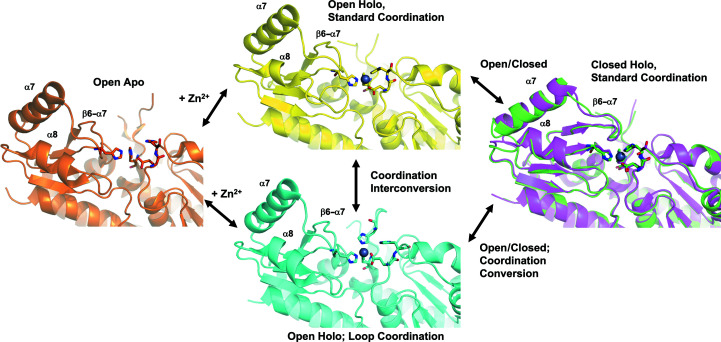
Structural landscape of ZnuA proteins. Representative structures of ZnuA in the open apo form (*Ec* ZnuA; Hesse *et al.*, 2019 ▸; orange; PDB entry 2ps3), the open holo form with coordination by His60 (*Ec* ZnuA; Hesse *et al.*, 2019 ▸; ‘Standard Coordination’; yellow; PDB entry 2prs chain *B*), the open holo form with coordination by loop residue His140 (*Se* ZnuA; Li & Jogl, 2007 ▸; ‘Loop Coordination’; cyan; PDB entry 2xqv chain *B*) and the closed holo form (*Ck* ZnuA; green; PDB entry 7rcj) aligned with *Ec* ZnuA (Hesse *et al.*, 2019 ▸; magenta, PDB entry 2prs chain *A*).

What is most remarkable about the structure of *Ck* ZnuA are the structural differences between it and *Se* ZnuA, despite the nearly 100% sequence identity between the proteins. Apart from changes in the loop region, which is disordered in both structures, there are only five very conservative amino-acid substitutions differentiating these proteins. Nevertheless, they adopt different zinc-coordination environments and the conformation of *Se* ZnuA is open while that of *Ck* ZnuA is closed. *Ck* ZnuA more closely resembles the closed conformation of holo *Ec* ZnuA, despite their lower sequence identity. Taken together, the structures of ZnuA from these three species suggest a diverse structural landscape and a potential pathway for zinc binding, as outlined below and in Fig. 3 ▸.

The only WT apo structure is that from *Ec* ZnuA and it exhibits an open conformation. Two zinc-bound structures exist in open forms with different coordination. The zinc in *Se* ZnuA is coordinated by His140 of the His-rich loop (Fig. 3 ▸, ‘Loop Coordination’) rather than the absolutely conserved His60 observed to coordinate in *Ec* ZnuA (Fig. 3 ▸, ‘Standard Coordination’). *Ck* ZnuA was solved in the holo form in a closed conformation very similar to that of the closed holo *Ec* ZnuA structure. These structures suggest a zinc-binding pathway whereby ZnuA acquires zinc in the open form first through a loop residue (His140 in *Se* ZnuA). His60 could then displace this residue, again in the open conformation, followed by closure of the β6–α7 loop and α7 and α8 helices to yield the closed, holo form. However, it is important to note that the loop is not required for high-affinity zinc binding in cluster A-I SBPs, as demonstrated here for *Ck* ZnuA and previously for several others (Wei *et al.*, 2007 ▸; Neupane *et al.*, 2017 ▸, 2019 ▸). Thus, Fig. 3 ▸ indicates simply the possibility of the interconversion of metal-coordination states rather than an obligate, directional process. Nevertheless, this conformational diversity among a closely related group of ZnuA proteins reveals a dynamic landscape that is likely to be functionally important and provides several potential targets for rational drug design.

## Supplementary Material

PDB reference: ZnuA from *Citrobacter koseri*, 7rcj

## Figures and Tables

**Table 1:** Macromolecule-production information

Source organism	*Citrobacter koseri*
DNA source	*Citrobacter koseri* genomic DNA
Forward primer	GCTCTCATATGATTAGTCGCATTATGTTACATAA
Reverse primer	ACTATGGTACCTTAATCTCCTTTCAGGCA
Cloning vector	pCDFDuet
Expression vector	pCDFDuet
Expression host	*Escherichia coli* BL21(DE3)
Complete amino-acid sequence of the construct produced	AVVASLKPLGFIASAIADGVTDTQVLLPDGASEHDYSLRPSDVKRLQGADLVVWIGPEMEAFMEKSVKNIPDGKQVTIAQLADVKPLLMKGADYNMHLWLSPEIARASAVAIHEKLVELMPQSRAKLDANLKDFEAQLAATDKQVGNELAPLKGKGYFVFHDAYGYYEKHYGLTPLGHFTVNPEIQPGAQRLHEIRTQLVEQKATCVFAEPQFRPAVVEAVARGTSVRMGTLDPLGTNIKLGKTSYSAFLNQLANQYASCLKG

**Table 2:** Crystallization

Method	Vapor diffusion
Plate	VDX
Temperature (K)	298
Protein concentration (mg ml^−1^)	18
Protein buffer	50 m*M* Tris pH 8.0, 150 m*M* NaCl
Reservoir composition	0.1 *M* sodium citrate pH 6.0, 0.05 *M* ammonium acetate, 1 m*M* hexatungstotellurate (TEW)
Drop volume and ratio	2 µl, 1:1 ratio
Reservoir volume (µl)	500

**Table 3:** Data collection and processing Values in parentheses are for the outer shell.

Diffraction source	Beamline 5.0.1, ALS
Wavelength (Å)	1.000
Temperature (K)	100
Detector	PILATUS3 6M 25 Hz
Crystal-to-detector distance (mm)	500
Rotation range per image (°)	1.0
Total rotation range (°)	200
Exposure time per image (s)	1.0
Space group	*P*2
*a*, *b*, *c* (Å)	122.25, 81.67, 126.56
α, β, γ (°)	90, 112.99, 90
Mosaicity (°)	0.31
Resolution range (Å)	47.5–3.15 (3.28–3.15)
Total No. of reflections	148165 (16672)
No. of unique reflections	39993 (4489)
Completeness (%)	99.8 (99.8)
Multiplicity	3.7 (3.7)
〈*I*/σ(*I*)〉	5.9 (0.8)†
*R*_r.i.m._	0.17 (1.30)
CC_1/2_	0.99 (0.34)
Overall *B* factor from Wilson plot (Å^2^)	89.3

† A cutoff of CC_1/2_ > 0.3 was used as data below the *I*/σ(*I*) cutoff were still valuable and improved the resolution, as discussed in the text.

**Table 4:** Structure refinement Values in parentheses are for the outer shell.

Resolution range (Å)	47.47–3.15 (3.232–3.150)
Completeness (%)	99.7
No. of reflections, working set	37978 (2771)
No. of reflections, test set	2006 (144)
Final *R*_cryst_	0.217 (0.365)
Final *R*_free_	0.254 (0.428)
No. of non-H atoms	
Protein	12237
Ion	6
Total	12243
R.m.s. deviations	
Bond lengths (Å)	0.012
Angles (°)	1.643
Average *B* factors (Å^2^)	
Protein	106.2
Ion	89.4
Ramachandran plot	
Favored regions (%)	91.2
Additionally allowed (%)	5.7
Outliers (%)	3.0
